# Tectorigenin Inhibits Glioblastoma Proliferation by G0/G1 Cell Cycle Arrest

**DOI:** 10.3390/medicina56120681

**Published:** 2020-12-10

**Authors:** Liang-Tsai Yeh, Li-Sung Hsu, Yi-Hsuan Chung, Chih-Jung Chen

**Affiliations:** 1 Department of Anesthesiology, Changhua Christian Hospital, Changhua 500, Taiwan; 68990@cch.org.tw; 2 Institute of Medicine, Chung Shan Medical University, Taichung 402, Taiwan; lshsu405@yahoo.com.tw; 3 Clinical Laboratory, Chung Shan Medical University Hospital, Taichung 402, Taiwan; 4 Institute of Biochemistry, Microbiology, and Immunology, Chung Shan Medical University, Taichung 402, Taiwan; jjlin159357@gmail.com; 5 School of Medicine, Chung Shan Medical University, Taichung 402, Taiwan; 6 Department of Pathology and Laboratory Medicine, Taichung Veterans General Hospital, Taichung 407, Taiwan

**Keywords:** glioblastoma, tectorigenin, molecular mechanism, in vitro model, cell cycle arrest

## Abstract

*Background and objectives:* Glioblastoma is one of the leading cancer-related causes of death of the brain region and has an average 5-year survival rate of less than 5%. The aim of this study was to investigate the effectiveness of tectorigenin, a naturally occurring flavonoid compound with anti-inflammatory, anti-oxidant, and anti-tumor properties, as a treatment for glioblastoma. A further goal was to use in vitro models to determine the underlying molecular mechanisms. *Materials and Methods:* Exposure to tectorigenin for 24 h dose-dependently reduced the viability of glioblastoma cells. *Results:* Significant cell cycle arrest at G0/G1 phase occurred in the presence of 200 and 300 µM tectorigenin. Treatment with tectorigenin clearly reduced the levels of phosphorylated retinoblastoma protein (p-RB) and decreased the expression of cyclin-dependent protein 4 (CDK4). Tectorigenin treatment also significantly enhanced the expression of p21, a CDK4 inhibitor. *Conclusions:* Collectively, our findings indicated that tectorigenin inhibited the proliferation of glioblastoma cells by cell cycle arrest at the G0/G1 phase.

## 1. Introduction

Glioblastoma multiforme (GBM), the most common malignant tumor in the brain region, has a high mortality and a very low 5-year survival rate (around 5%) [1]. In general, the treatment of GBM depends on resection, chemotherapy, and radiotherapy [1]. However, because GBM has a rapid growth, high invasion ability, and pronounced drug resistance, its prognosis is very poor. New GBM therapeutic agents with less toxicity and more effectiveness are therefore urgently needed.

One possible therapeutic agent is tectorigenin, the active flavonoid in a Chinese medicine extract [2]. This compound shows several important properties, ranging from anti-inflammation and antioxidant to anti-tumor activities. For example, Zhang et al. [3] demonstrated that tectorigenin prevented H_2_O_2_-induced cell death in Chinese hamster lung fibroblast (V79-4) cells by increasing the activities of catalase, extracellular signal-regulated kinase (ERK), and nuclear factor kappa-light-chain-enhancer of activated B cells (NF-kB). Similarly, tectorigenin treatment reduced lipopolysaccharide-induced neuroinflammation by downregulation of the NFkB and Jun N-terminal kinase (JNK)/ERK pathways [4]. Tectorigenin treatment also enhanced the expression of CD14 and CD66b in HL-60 leukemia cells and promoted their differentiation into monocytes/macrophages and granulocytes [5]. Tectorigenin also reduced the expression of prostate-derived ETS factor (PDEF), prostate specific antigen(PSA), and insulin-like growth factor-1 (IGF-1) receptor mRNA in LNCaP prostate cancer cells [6]. Notably, tectorigenin treatment of HL-60 cells blocked the activities of epidermal growth factor receptor (EGFR), repressed the expression of Bcl-2, and eventually triggered apoptosis [5].

A similar triggering of apoptosis was also reported in hepatocellular carcinoma cells treated with tectorigenin, and this apparently occurred due to elevation of levels of reactive oxygen species (ROS), disruption of mitochondrial function, and activation of the caspase cascade [7]. Yang et al. demonstrated that tectorigenin acted synergistically with paclitaxel to induce apoptosis in drug-resistant ovarian cancer cells by inhibition of the AKT [a serine/threonine-specific protein kinase, also known as Protein kinase B (PKB)] pathway and downregulation of NFkB downstream targets [8]. In cocultures of A549 lung cancer and THP1 cells, tectorigenin suppressed the expression of apoptosis-related genes (including those responsible for pro-inflammatory cytokine production, such as interleukin-6 and tumor necrosis factor alpha), inhibited the expression of snail, and upregulated E-cadherin expression [9]. The aim of the present study was to investigate whether tectorigenin has similar cell cycle effects in glioblastoma cells. 

## 2. Materials and Methods

### 2.1. Cell Culture 

Human GBM-8401 and GBM-8901 glioblastoma cancer cells were obtained from American Type Culture Collection (Manassas, VA, USA) and cultured in RPMI medium supplemented with 10% fetal bovine serum (FBS), 100 U/mL penicillin, and 100 μg/mL streptomycin at 37 °C in a humidified atmosphere with 5% CO_2_.

### 2.2. MTT [3-(4,5-dimethylthiazol-2-yl)-2,5-diphenyltetrazolium bromide] Assay 

The GBM-8401 and GBM-8901 cells were seeded in 24-well plates at a density of 5 × 10^4^ cells/mL and exposed to 0, 25, 50, 100, 200, or 300 µM tectorigenin for 24 h. After the treatment, the culture medium was replaced with fresh medium containing 5.0 g/L MTT and the cells were incubated at 37 °C for an additional 2 h. The cells were pelleted by centrifugation, the sediments were dissolved in 1 mL isopropanol, and the absorbance at 563 nm was determined. The relative viability rate was determined based on the absorbance at 563 nm of each treatment compared with vehicle-treated cells.

### 2.3. Flow Cytometry Analysis

The GBM-8401 and GBM-8901 cells were treated with the indicated concentration of tectorigenin for 24 h. The cells were then harvested, fixed with 70% ethanol overnight, and stained with 50 μg/mL propidium iodide (PI) in the dark. The cell cycle distribution percentages were analyzed by flow cytometry with a BD Biosciences FACScan system using the CellQuest^TM^ Pro software.

### 2.4. Western Blot Analysis 

Tectorigenin-treated GBM-8401 cells were lysed in 150 μL radioimmunoprecipitation assay (RIPA) lysis buffer (Thermo Fisher Scientific, Inc., Waltham, MA, USA). The lysates were centrifuged at 14,000× *g* for 10 min and the supernatants were collected. A total of 50 μg protein was separated on a 10% polyacrylamide gel and transferred onto a nitrocellulose membrane (Merck Millipore, Darmstadt, Germany). The membrane was blocked with phosphate buffered saline (PBS) containing 0.5% non-fat milk for 1 h at room temperature. After washing with PBS containing 0.1% Tween-20 (PBST), the membrane was incubated with primary antibodies at 4 °C overnight. The primary antibodies included phosphorylated RB (AP0089, Abclonal Com.), cyclin dependent kinase 4 (CDK4) (A0366, Abclonal Com.), p21 (A1483), and p27 (A16633, Abclonal Com.). The membrane was then washed with PBST and reacted with horseradish peroxidase-conjugated secondary antibody (Santa Cruz Biotechnology, Inc., Dallas, TX, USA). After extensive washing of the membrane with PBST, the positive signal was determined by enhanced chemiluminescence (GE Healthcare Life Sciences, Chalfont, UK). Glyceraldehyde-3-phosphate dehydrogenase (GAPDH; 10494-1, Proteintech Com.) expression was used as an internal control.

### 2.5. Statistical Analysis 

The data represent the mean ± standard deviation of three independent experiments and were evaluated by Student’s t test using SPSS 14.0 software (SPSS, Inc., Chicago, IL, USA). A value of *p* < 0.05 was considered statistically significant.

## 3. Results

### 3.1. Tectorigenin Inhibited Cell Proliferation and Induced G0/G1 Arrest in Glioblastoma Cells

**Figure 1:**
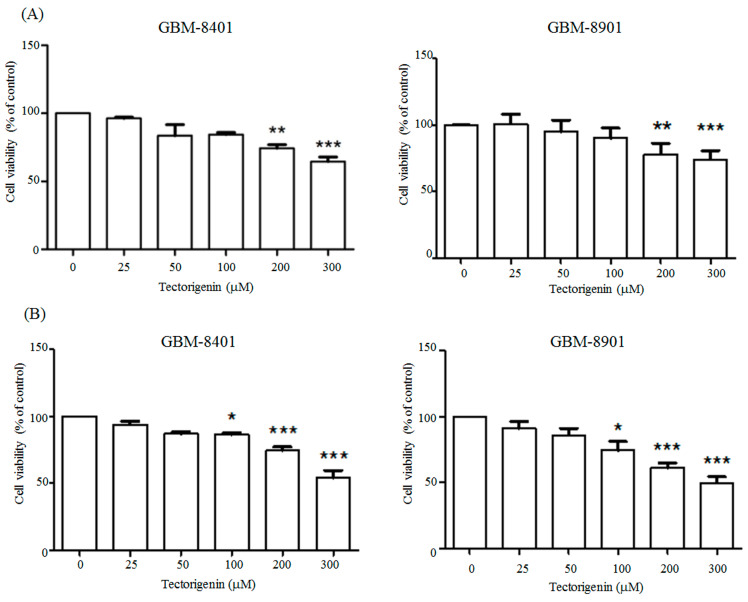
Tectorigenin reduced cell viability in glioblastoma cells. Glioblastoma GBM-8401 and GBM-8901 cells were treated with 0, 25, 50, 100, 200, and 300 μM tectorigenin for (**A**) 24 h and (**B**) 48 h. Cell viability was detected with the MTT assay. Data represent means ± standard deviation obtained from at least three independent experiments. *: *p* < 0.05; **: *p* < 0.01; ***: *p* < 0.001.

We assessed the cytotoxicity of tectorigenin in the GBM cells by treating glioblastoma GBM-8401 cells with 0, 25, 50, 100, 200, or 300 µM tectorigenin for 24 h and then determining the cell viability with the MTT assay. The cell viability was significantly reduced to 74% and 65% by 200 and 300 µM tectorigenin, respectively (Figure 1A). Similarly, the cell viability of the GBM-8901 cells was reduced to 78% and 74% by a 24 h treatment with 200 and 300 μM tectorigenin, respectively (Figure 1A). Treatment with 0, 25, 50, 100, 200, or 300 µM tectorigenin for 48 h gave cell viability values of 93%, 87%, 86%, 74%, and 54% for GBM-8401 cells and 91%, 86%, 74%, 61%,and 50% for GBM-8901 cells, respectively (Figure 1B).

**Figure 2:**
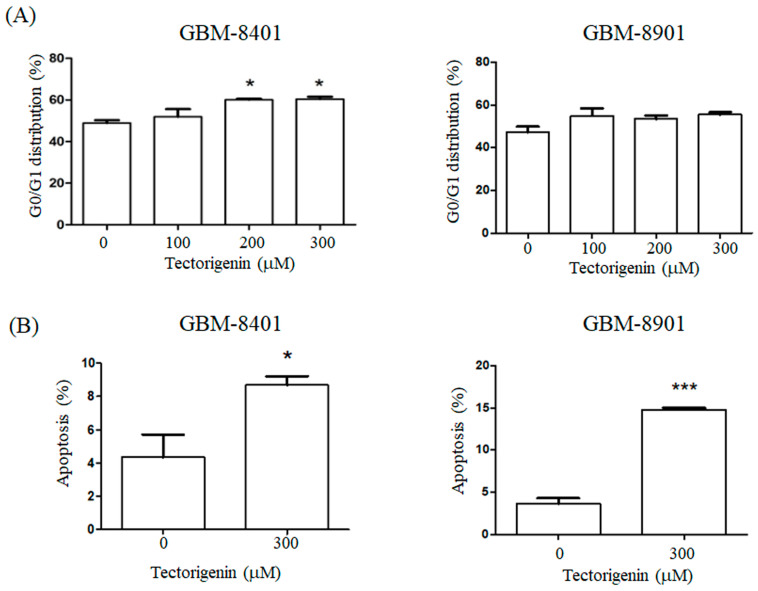
Tectorigenin induced (**A**) G0/G1 arrest and (**B**) apoptosis in glioblastoma cells. (**A**) Glioblastoma GBM-8401 and GBM-8901 cells were treated with 100, 200, and 300 μM tectorigenin for 24 h. The G0/G1 cell cycle distribution was detected by flow cytometry analysis. (**B**) GBM-8401and GBM-8901 cells were treated with 300 μM tectorigenin for 48 h and apoptosis was analyzed by flow cytometry. Data represent means ± standard deviation obtained from at least three independent experiments. *: *p* < 0.05. ***: *p* < 0.001.

We also used flow cytometry to examine the effects of tectorigenin on the cell cycle progression in GBM cells. Tectorigenin at 200 and 300 µM significantly increased the percentage of GBM-8401 cells in the G0/G1 phase to 60.0% and 60.4%, respectively, compared to 48.9% in untreated control cells (Figure 2A). Conversely, no overt alteration in the G0/G1 population was found for the GBM-8901 cells in response to tectorigenin treatment (Figure 2A).Treatment with 300 μM tectorigenin for 48 h induced apoptosis in both GBM-8401 and GBM-8901 cells (Figure 2A,B). 

### 3.2. Tectorigenin Altered the Status of Cyclin-Dependent Protein Kinase 4 (CDK4) and Phosphorylated Retinoblastoma Protein (RB)

**Figure 3:**
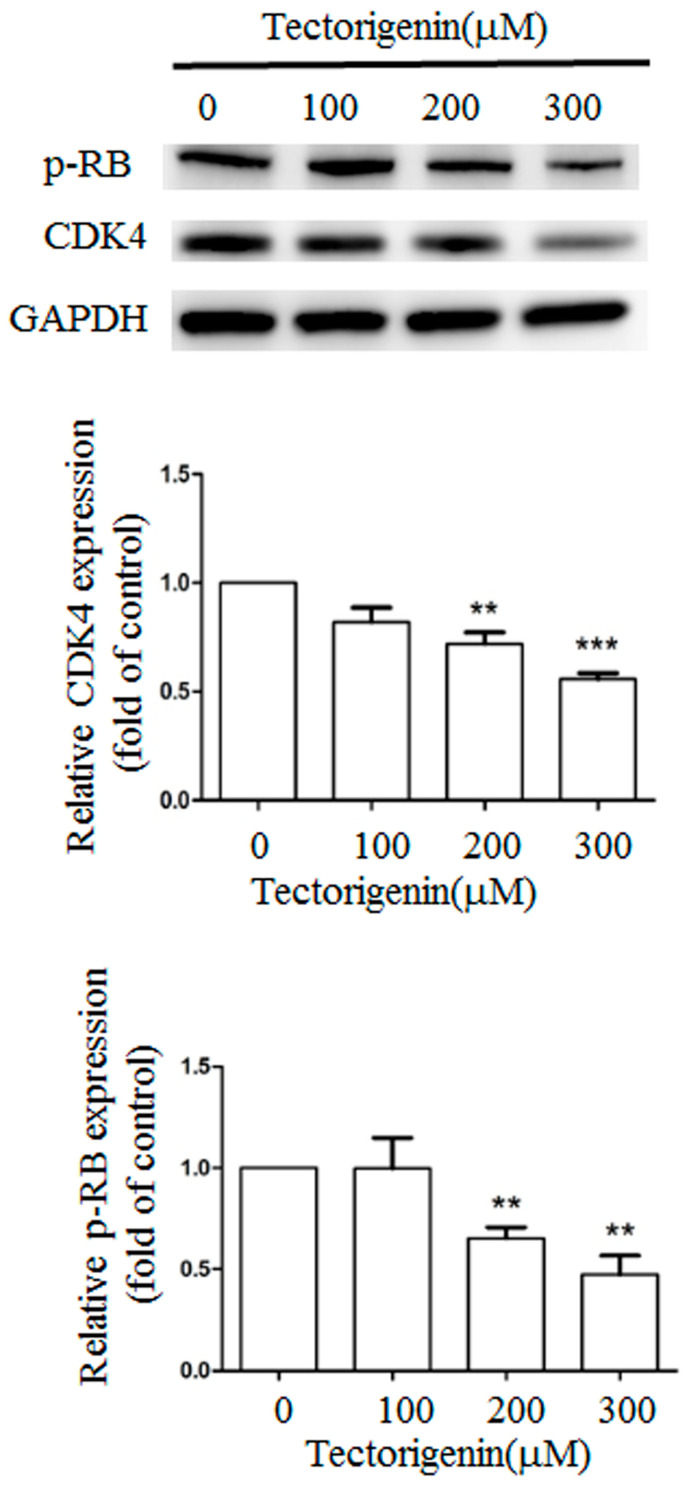
Tectorigenin altered the expression of CDK4 and phosphorylated RB proteins. Glioblastoma GBM-8401 cells were treated with 100, 200, and 300 μM tectorigenin for 24 h. Cell lysates were purified and subjected to western blot analysis. Quantitative data represent means ± standard deviation from three independent experiments. **: *p* < 0.01; ***: *p* < 0.001 compared with control group.

CDK4 and phosphorylated RB play critical roles in cell cycle progression from the G0/G1 to the S phase. We determined whether tectorigenin influenced the CDK4 and phosphorylated RB levels by western blotting. Tectorigenin at 100, 200, and 300 µM markedly reduced the expression of CDK4 to 82.0%, 72.0%, and 55.7% of the control level, respectively, and the expression of phosphorylated RB to 99.7%, 65.3%, and 47.3%, respectively (Figure 3). 

### 3.3. Tectorigenin Treatment Increased the Expression of p21 but Not p27

**Figure 4:**
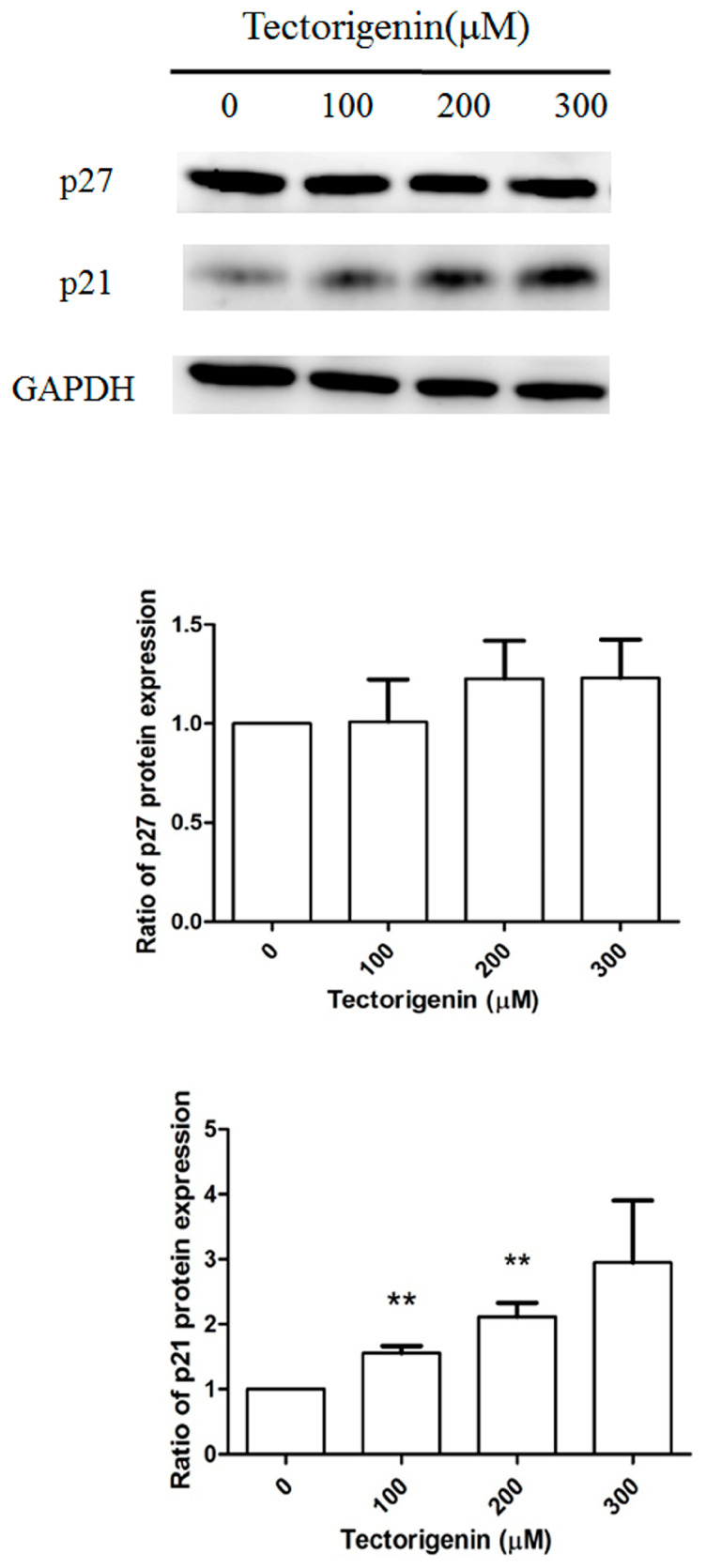
Tectorigenin altered the expression of cell cycle-related proteins. Glioblastoma GBM-8401 cells were treated with 100, 200, and 300 μM tectorigenin for 24 h. Cell lysates were purified and subjected to western blot analysis. Quantitative data represent means ± standard deviation from three independent experiments. **: *p* < 0.01 compared with control group.

The effects of tectorigenin on the expression of CDK inhibitors was further examined by western blotting. As shown in Figure 4, treatment with 100, 200, and 300 μM tectorigenin increased p21 expression by 1.46-fold, 1.69-fold, and 1.89-fold, respectively, compared with vehicle-treated control cells. By contrast, p27 expression was not affected by tectorigenin treatment at any of the tested concentrations.

## 4. Discussion

GBM is the most commonly encountered malignant brain tumor. Unfortunately, due to its late diagnosis and high metastatic potential, GBM has a 5-year survival rate of only around 5% [1]. Here, we examined whether tectorigenin, which exhibits anti-inflammation, anti-oxidant, and anti-tumor activities, might be potentially therapeutic as a GBM treatment. Specifically, in this study, we highlighted the underlying mechanisms that lead to tectorigenin suppression of GBM cell proliferation.

Increased cell cycle progression results in enhanced cell proliferation and plays an important role in cancer development [10]. Many reports have shown that flavonoids like tectorigenin exert their anti-cancer effects by triggering cell cycle arrest at the G0/G1 or G2/M checkpoints. For example, jaceosidin at 100 µM significantly enhanced the G2/M arrest of glioblastoma U87 cells [11], whereas licochalcone A triggered both G0/G1 and G2/M arrest in glioblastoma U87cells in vitro and in vivo [12]. Fang et al. [13] demonstrated an increased percentage of COR-L23 cells in the G2/M phase in proportion to the tectorigenin concentration (from 50 to 400 µM). Another previous report also demonstrated a significantly increased G0/G1 phase population in breast cancer MDA-MB-231 cells treated with 100 or 200 µM tectorigenin for 24 h [14]. In agreement with these previous studies, we showed that tectorigenin at 200 and 300 µM clearly induced G0/G1 cell cycle arrest in glioblastoma GBM-8401 cells. 

Two well-known promoters of cell cycle progression are CDK and its associated cyclin [15,16]. CDK4 forms a complex with cyclin D to phosphorylate RB, and this then triggers the disassociation of RB and E2F to promote cell cycle transition to the S phase [15]. Previous studies have shown that the expression of CDK4 at the mRNA level was significantly increased in anaplastic astrocytoma and glioblastoma tumors when compared to adjacent normal brain tissues [17]. Ruano et al. [18] demonstrated a correlation between the amplification of CDK4 and a worse survival rate in patients with malignant astrocytic gliomas. Lien et al. [19] showed that the flavonoid nobiletin suppressed the proliferation of human U87 and Hs683 glioma cells by downregulating the expression of CDK2, CDK4, and cyclin D1. Similarly, the flavonoid phloretin increased the production of reactive oxygen species and dose-dependently decreased the expression of CDK2, CDK4, and cyclin D to ultimately inhibit the proliferation of human glioblastoma cells [20]. Our findings with tectorigenin, another flavonoid, are in line with these previous observations, as tectorigenin repressed CDK4 expression, reduced the phosphorylation of RB protein, and eventually diminished the proliferation of human glioblastoma GBM-8401 cells.

CDK inhibitors are also known to play an important negative regulatory role in cell cycle progression [21]. Disruption of the CDK4/cyclin D complex promotes the expression of p16, p21, and p27 and triggers cell cycle arrest at the G0/G1 phase [21]. Similarly, expression of p27, p21, and p53 block the activity of the CDK2/cyclin E complex to cause G2/M phase arrest [21]. In the present study, tectorigenin treatment of glioblastoma cells markedly increased p21 expression. Lin et al. [22] demonstrated that another flavonoid, hispidulin, induced G1 cell cycle arrest in GBM-8401 and GBM-8901 cells by enhancing p21 and p53 expression. Similarly, wogonin, a flavonoid from *Scutellaria baicalensis*, upregulated p53 and p21 expression and then increased the population of glioblastoma cells in the G0/G1 phase [23]. Another flavonoid, icariside II, attenuated the phosphorylation of forkhead box O3a (FOXO3a), increased the transcription of p21 and p27, and then caused cell cycle arrest in glioblastoma cells [24]. 

In the present study, we demonstrated that the flavonoid tectorigenin promoted p21 expression, decreased the levels of CDK4 and phosphorylated RB protein, triggered G0/G1 cell cycle arrest, and eventually attenuated the proliferation of glioblastoma cells. These findings suggest that tectorigenin may have potential therapeutic promise as an anti-glioblastoma agent.
